# Revaluing unmanaged forests for climate change mitigation

**DOI:** 10.1186/1750-0680-7-11

**Published:** 2012-11-14

**Authors:** Joachim Krug, Michael Koehl, Dierk Kownatzki

**Affiliations:** 1 Johann Heinrich von Thuenen-Institute (vTI), Federal Research Institute for Rural Areas, Forestry and Fisheries, Institute for World Forestry, Leuschnerstr. 91, 21031, Hamburg, Germany

**Keywords:** Unmanaged forests, Climate change mitigation, CO_2_ sequestration, Forest management, Substitution

## Abstract

**Background:**

Unmanaged or old-growth forests are of paramount importance for carbon sequestration and thus for the mitigation of climate change among further implications, e.g. biodiversity aspects. Still, the importance of those forests for climate change mitigation compared to managed forests is under controversial debate. We evaluate the adequacy of referring to CO_2_ flux measurements alone and include external impacts on growth (nitrogen immissions, increasing temperatures, CO_2_ enrichment, changed precipitation patterns) for an evaluation of central European forests in this context.

**Results:**

We deduce that the use of CO_2_ flux measurements alone does not allow conclusions on a superiority of unmanaged to managed forests for mitigation goals. This is based on the critical consideration of uncertainties and the application of system boundaries. Furthermore, the consideration of wood products for material and energetic substitution obviously overrules the mitigation potential of unmanaged forests. Moreover, impacts of nitrogen immissions, CO_2_ enrichment of the atmosphere, increasing temperatures and changed precipitation patterns obviously lead to a meaningful increase in growth, even in forests of higher age.

**Conclusions:**

An impact of unmanaged forests on climate change mitigation cannot be valued by CO_2_ flux measurements alone. Further research is needed on cause and effect relationships between management practices and carbon stocks in different compartments of forest ecosystems in order to account for human-induced changes. Unexpected growth rates in old-growth forests – managed or not – can obviously be related to external impacts and additionally to management impacts. This should lead to the reconsideration of forest management strategies.

## Background

Following the FAO definition, “unmanaged forests” are characterized by the lack in formal management, e.g. in the preference of natural development of forests for nature conservation purposes [1]. Forest Europe, UNECE and FAO reported 1.476 million hectares of the forest area in central Europe as “undisturbed by man” in 2012 [2]. In core areas of German national parks and German forest nature reserves at minimum 0.1 million hectares of unmanaged and mainly old-growth forests can be found in which logging and thinning are not allowed [3]. Although these strictly protected areas represent less than one percent of the total forest area in Germany, they meaningfully contribute to carbon storage in forests. However, unmanaged forests are not part of the reporting on carbon stocks in forests under the Kyoto Protocol (but to regular UNFCCC reporting) as only directly human-induced carbon sequestration is accountable here. Verified data on carbon stocks and carbon stock changes in unmanaged forests are limited and mostly restricted to short-term monitoring as phases of either carbon uptake or carbon loss may alter as well as the actual amount of their carbon stocks changes within the life-cycle.

Forest ecosystems provide an important contribution to climate mitigation by sequestering and storing carbon. The sequestration of atmospheric carbon dioxide takes place by photosynthesis and the conversion to biomass carbon and subsequent carbon fluxes: the storage of carbon in above- and below-ground (dead and living) biomass and related soil (organic) carbon pools. Naturally, decay complements growing processes and releases carbon again, mainly as carbon dioxide. Little is known about degeneration processes and related CO_2_ fluxes of more or less even aged forests which have been abandoned from management and enter into over-matured phases: those forests can be supposed to become a principal CO_2_ source. In contrast to unmanaged forests, managed forests provide the potential for extending that cycle of carbon sequestration and storage by timber utilisation. The carbon pool is enlarged by harvested wood products (HWPs) and their potential of substituting fossil fuel or energy-intensive materials, [4-6].

In view of climate change there is a general agreement to preserve and maintain carbon pools of unmanaged forests, as those sustain substantial carbon pools. Saatchi et al. assessed forest carbon stocks for 75 developing countries and estimate the total biomass carbon stock of forests to be 247 Gt C, with 193 Gt C stored in aboveground and 54 Gt C stored in belowground biomass [7]. Carbon pools of unmanaged forests are often larger than those of managed forest stands under similar biophysical conditions [8].

The sequestration of carbon dioxide emissions, i.e. resulting from fossil fuel use, is a core aspect for mitigating future climate change [9]. In this view, next to carbon storage in forest biomass, any sequestration of atmospheric carbon dioxide by photosynthesis and timber growth additional to decay processes is of paramount importance. This is generally accepted for managed forest stands and irrespective optimisation measures are intensively discussed, e.g. by [10-13]. There is however an ongoing controversial dispute about the ability and magnitude of carbon dioxide sequestration exceeding decay processes in unmanaged forests. Especially old-growth forests are subject of the debates which often result in conflicting points of view on the mitigation potential of unmanaged versus managed forests.

Another major problem is the definition of the system boundaries; while the “ecosystem view” is limited to forests, the “forest sector view” extends to the storage and substitution effects of harvested wood products. Recommendations derived from both views can result in contradictory political advice and proposals for allocating incentives for the mitigation of climate change. This could, for example, be of importance in discussions on the implementation of the German Government’s “National Strategy on Biological Diversity”, where one aim is that “By 2020 forests with natural forest development account for 5% of the wooded area” [14].

## Methods

This study provides a critical appraisal of published research results on the relevance of unmanaged forests for carbon dioxide sequestration in central Europe that underlie a well-founded view on the mitigation potential of unmanaged forests and corresponding recommendations for the political negotiation processes on climate change mitigation.

Due to the substantial differences between biomes, boreal and Mediterranean regions are intentionally excluded for this study. While temperate forests are distinguished by a moderate climate with a growing season of 140–200 days during 4–6 frost-free months and precipitation (75–150 cm) that is distributed evenly throughout the year, the climate of boreal forest is characterized by short, moist, and moderately warm summers and long, cold, and dry winters. The differences in climate result in distinct differences in growth patterns and biogeochemical processes. Where the reference to “temperate and boreal forests” indicates that those effects are not separated, results cannot simply be conveyed to statements on central European conditions. Such reference, as observed in some publications about carbon dioxide sequestration in central Europe (see [15-17]), will lead to misleading interpretations.

According to Schuck et al. there is no common agreement for a definition of “unmanaged forests” [18]. In a more general sense it describes forests which have been excluded from forest management by legal status and left to develop undisturbed by man while tolerating all kinds of natural disturbances [18]. Although the duration of the abandonment of any management impact such as harvesting or thinning is often unknown, Wirth et al. and Knohl et al. define “old-growth forests” as forests without any management activity for at least 250 years – which actually excludes most the forested area in central Europe [19,20]. In our study we further excluded references to sites on steep slopes, forest stands on organic soils, and forest patches less than one hectare. Forest stands on those sites might be unmanaged, but the specific site conditions would render the comparison with forests under management difficult.

The study concerns the research results from closely related research projects such as “CarboEurope-IP” [21] and “Potential and dynamics of carbon sequestration in forests and timber products (CSWH)” [22] and further results published in peer-reviewed journals. The majority of the analysed research results refers to deciduous forests. It must be considered that consequences of utilisation abandonment differ in coniferous forests and would impose higher risks in terms of calamities and stand stability.

## Results and discussion

### Consideration of CO_2_ flux measurements for determining sequestration rates and relating those to biological processes

The evaluation of eddy-covariance based CO_2_ flux measurements is widely used to describe the net ecosystem productivity (NEP) and where soil respiration assessments are considered the net primary productivity (NPP) [16,20,23-26]. In this context, the term “productivity” is to be understood as a component in the carbon cycle [21].

Luyssaert et al. describe gas flux measurements from 519 global plot studies, which provided estimates of sequestration rates of about 2.4 ±0.8 tC/ha/yr in forest stands older than 200 years [17]. Those results could be used to falsify the “long-standing view that old-growth forests are carbon neutral” [17] - the equilibrium hypothesis that was originally established by Odum in 1969 [27]. The line of argumentation presented by Luyssaert et al. does not explain the process of carbon sequestration itself, i.e. which pools continue to be increased [17]. In fact, Luyssaert et al. present a model-based assumption that the averaged sequestration rate of 2.4 ±0.8 tC/ha/yr is to be divided in 0.4 ±0.1 tC/ha/yr (measurable) stem biomass, 0.7 ±0.2 tC/ha/yr (measurable) coarse wood debris, and imply the remaining 1.3 ±0.8 tC/ha/yr from gas flux measurements has to be stored in roots and soil organic matter. Based on this difference calculation Luyssaert et al. conclude that old-growth forests sequester carbon in root and soil organic matter beyond the equilibrium of biomass growth and biomass decay [17].

It is not our aim to put the approaches and results of gas flux measurements into question, but we critically challenge the respective sufficiency of a difference calculation for drawing conclusions on the mitigation potential of old-growth forests. Results from gas flux measurement from above or within the crown layer for the determination of the NEP must be viewed from a wider angle than simply establishing a numerical balance that assigns the residual differences from measured quantities to e.g. soil organic carbon. Uncertainties of current flux measurements, measured biomass components and applied calculation methods render further studies for conclusions of carbon flux from atmospheric carbon to biomass carbon and soil carbon and related carbon sequestration potentials of old-growth forests necessary (compare Luyssaert et al. [17,24]). This holds especially true where further implications such as the impact of CO_2_ fertilisation and nitrogen depositions are drawn.

Luyssaert et al. themselves advise caution to avoid misinterpretation of their results by indicating “there is some degree of age-related decline in NPP beyond 80 years of age (…), and temperate and boreal forests both show a consistent pattern of declining NPP beyond an early maximum (…) when analysed separately” [17]. In a later publication on the European forest carbon balance Luyssaert et al. notice large uncertainties related to the “estimates of the fate of carbon inputs via NPP” [24]. They reduce the fraction of postulated forest soil carbon: “new estimates of the long-term carbon forest sink (…) of EU-25 forests …” amount to 0.75 ±0.2 tC/ha/yr, and “inventory-based assessments and assumptions suggest…” that about 0.22 tC/ha/yr is sequestered in forest soils. These recently published figures reduce previous results (1.3 ±0.8 tC/ha/yr) by more than 80% [17] and [24].

Still, the main intention of Luyssaert et al. [17] was not to falsify the old-growth equilibrium hypothesis of Odum [27], but to point out the importance of a consideration of old-growth forests within greenhouse gas monitoring. Of major concern is the reference made by other authors to the results presented by Luyssaert et al., whose data are uncritically adopted for estimating the potential carbon sequestration in old-growth forests. For example, NABU [28] and Freibauer et al. [15] used those data to explain that the “biomass in temperate and boreal forest increases exponentially by age” and that “old forests, by that, are more efficient carbon-sinks than young forests” (translated from Freibauer et al. [15]).

Luyssaert et al. conclude in accordance with other literature that old-growth forests do reach equilibrium between growth and mortality of tree biomass [17]. Based on their findings old-growth or unmanaged forests can provide higher sequestration rates than managed forests where large amounts of dead organic matter are transferred to the soil carbon pools. Considering the loss of forest biomass by timber harvesting and the potentially disturbing impact of harvesting activities on the forest floor, those results are evident. However, inferring from CO_2_ flux measurements to soil carbon sequestration rates and especially the quantification of carbon sequestration rates of unmanaged forests must be considered to be critical. In line with the argumentation given by Luyssaert et al. [17,24], anticipating NPP for the quantification of soil carbon sequestration rates and the subsequent productivity of old-growth forest stands is not straightforward. Drawing conclusions from NPP on the productivity of forest stands needs to take into account the underlying uncertainties of carbon fluxes between different pools and should be supported by *insitu* measurements and related models.

Comparable caution must be taken when soil carbon inventories are used for complementing CO_2_ flux measures: especially soil carbon inventories are related to high uncertainties ( [29,30]) and allow only limited conclusions when the accuracy of estimates is ignored. Luyssaert et al. point out that “… large uncertainty remains concerning the drivers and future of the soil organic carbon” [24]. Schulze et al. state that “the soil in the unmanaged forests also contained more carbon, although it is not clear if this difference is caused by the absence of timber extraction, by difference in the historical management, or by small differences in soil properties” [21].

### Changing growing conditions for managed and unmanaged forests resulting in additional carbon uptake

Besides direct management impacts tree growth is influenced by the effect of changing climate conditions, of CO_2_ fertilisation and of nitrogen depositions from the atmosphere (de Vries et al. [31]; Laubhann et al. [32]). Burschel and Huss describe for central European regions that the interrelations between forest management and soil are already superimposed by anthropogenic immissions, predominantly nitrogen depositions, and increased atmospheric CO_2_ concentrations [33]. According to Burschel and Huss those impacts overcome and often over-compensate historical nutrient deficiencies. They show e.g. yields of Scots Pine (*Pinus sylvestris*) on rather poor sites (normally representing yield class IV according to [34]) that by far exceed expected yields of best sites (belonging to yield class I according to [34]).

This is further supported by the RECOGNITION project: Kahle et al. state that of the possible causes of observed growth increase in European forests during the last decades (increasing atmospheric carbon dioxide concentration, improved temperature and precipitation climate, increasing nitrogen deposition and better management), the major cause is attributed to increasing nitrogen depositions, while direct temperature effects and increasing atmospheric carbon dioxide concentration are likely to become important determinants of forest growth in future [35].

Körner et al. explain that an increased CO_2_ concentration solely would not cause increased growth, which leads to the assumption that other limiting factors like nutrient deficiencies are predominant [36]. Further studies support those observations: Magnani et al. find a positive relationship between the average net ecosystem production (NEP) and wet N deposition, which state that “net carbon sequestration of temperate […] forests is clearly driven by nitrogen” [37]. More specifically the authors state that “…we are actually controlling the carbon balance of our forests by the inadvertent addition of nitrogen fertilizer. We believe that forests respond to temperature largely because microbial activity increases and the soil organic matter decomposes more rapidly. This releases more nutrients which are needed for tree growth. By adding extra nitrogen through fertilizer or air pollution we throw a system which was previously in equilibrium out of balance and it responds by greater growth, increasing the amount of carbon stored in the wood and the soil”. Milne and Van Oijen as well confirm that “…main driver of increased forest growth in the 20th century has been increased nitrogen deposition” [38]. Laubhann et al. conclude that “an increase of 1 kg N/ha/yr corresponds to an increase in basal area increment between 1.20% and 1.49% depending on species [32]. Considering an average total carbon uptake for European forests near 1730 kg per hectare and year, this implies an estimated sequestration of approximately 21–26 kg carbon per kg nitrogen deposition”. This impact is relevant to managed as well as unmanaged forests and could explain observed deviations from past growth rates as described by Spiecker et al. [39]. While Magnani et al. discuss the magnitude of such an impact on changed growing conditions [37], Mund et al. separate such an impact from management effects like early thinning [40]. Mund et al. observe for example an average increment in stem volume at stand level which was about 90% higher compared to predictions of common yield tables [34,41] on stand level for even-aged experimental stands at ages from 16 to 142 year old sample trees. Therefore, they conclude that the growth of Norway spruce (*Picea abies*) is significantly higher than indicated in common yield tables representing growing conditions from before 1960.

With a similar point of view, Schulze et al. claim that “nitrogen fertilization and possibly the effects of increased atmospheric CO_2_ concentration and higher temperatures resulting from climate change all suggest we should expect unmanaged forests to continue absorbing carbon” [21]. They stated further that “… at any moment the rate of carbon uptake at a particular site will depend on the age composition and density of the stand. Forest management also influences growth rates by controlling stand density, and management practises have been changing over recent decades. Additional factors that might be influencing forest growth rate are increased temperature and carbon dioxide concentration, or nitrogen deposition from the atmosphere.”

In summary, changing growing conditions are obviously correlated to an increase of carbon sequestration: slightly increased temperatures, possibly an increased CO_2_ concentration and obviously nitrogen fertilisation do provide more favourable growing conditions for temperate forests. Vetter et al. referred to a combination of model simulations and forest inventories of coniferous forests in Thuringia (a federal state of Germany) and found that “… older forests had the strongest relative increase in carbon sequestration …” by comparing their growth rates to a baseline scenario [42]. Based on their model assumptions up to 33% of the carbon sink can be related to environmental changes during the past century, i.e. warmer temperatures, fertilizing effects of increased CO_2_ and nitrogen deposition [42].

Those environmental changes are long-range and transboundary in nature and thus have impacts on forest stands irrespective of their management status. Additional carbon uptake cannot be attributed to unmanaged forest only. It is widely accepted that the growth of managed forests is accelerated by recent environmental changes [21,32,37,39,40,42,43]. Increased biomass growth is a phenomenon observed in the entire central European forests and thus does not qualify to stipulate any additional benefits of unmanaged forests in terms of increased carbon sequestration, compared to managed forests.

Apart from positive or negative impacts on growth, changing environmental conditions also influence forest structures, disturbance regimes, species spectrum, humus turnover, soil carbon storage etc. – here the difference between managed and unmanaged forests might be larger, especially when it is considered that forest management offers the possibility of active adaptation.

### Accounting of additional growth under the requirements of UNFCCC and the Kyoto Protocol

COP 7 (Marrakesh, October/November 2001) adopted fundamental decisions on the accounting of land use, land use change and forestry (LULUCF) and related issues. The rules for LULUCF activities, agreed on as part of the so-called Marrakesh Accords (Decision 11/CP.7, IPCC 2002 [8]), provide detailed specifications to estimate, measure, monitor, and report changes in carbon stocks and anthropogenic greenhouse gas emissions by sources and removals by sinks resulting from LULUCF activities under Article 3, paragraphs 3 and 4, and Articles 6 and 12 of the Kyoto Protocol. Within those, it was explicitly stated that naturally-occurring removals, including removals as a consequence of indirect anthropogenic effects “such as those from carbon dioxide fertilization and nitrogen deposition”, should be excluded and “factored out”.

The here discussed additional or unexpected growth observed in unmanaged forests is obviously referred to such “indirect anthropogenic effects” by the cited literature. Consequently, these measurable positive effects on additional carbon sequestration are not accountable under the Kyoto Protocol [21,44].

As similar indirect anthropogenic effects on unmanaged forests are to be considered for managed forest stands as well, the importance of assessing and evaluating the direct human-induced impact on forest growth and carbon sequestration is obvious. Human-induced changes for increased carbon sequestration, e.g. caused by different management practises, the utilisation of adapted species or the renunciation of harmful practises (like forest litter utilisation, whole-tree harvesting etc.), are to be separated from non human-induced changes.

In the decision 16/CMP.1 (the so-called Accra Accounting Options [45]) the integration of a cap or discount factor on accountable credits and debits was adopted to limit indirect human impacts, among further aims. Both, the discount factor and the cap, were initially proposed at 15% of the reported credits and debits from LULUCF. This was a rather pragmatic approach based on the estimation that about 15% of the increment of forest stands was preferable to human-induced impacts [45]. The level of 15% was adopted to approximate the human impact on residual terrestrial uptake as “best estimate under a conservative approach” for European forests (Fischlin 2011, pers. com.). Another method to separate human-induced from non human-induced impacts for accountable credits and debits from LULUCF is referring to the difference of emissions between two periods [44]. Here the assumption is made that non-human-induced impacts on the reference period remains at similar levels for the accounting period, while any change between the two periods is assigned to human-induced impacts. Still, both accounting approaches cannot reflect the actual magnitude of the direct human-induced impact on forest growth and carbon sequestration. This again reflects the difficulties and uncertainties by attempting to separate human-induced from non-human-induced impacts on forest growth. Lindner et al. point out “when carbon stocks are compared between unmanaged and managed forest stands, unmanaged stands typically show higher stocks. (…) In order to account for human-induced changes, a clear cause and effect relationship between management practices and carbon stocks in different compartments of the ecosystem is required” [46].

In addition to that, an enlargement of the definition of the system boundaries from the “ecosystem view” to the “forest sector view” extends mitigation potentials beyond carbon storage effects of forests. Since the accountability of harvested wood products was proposed for a second commitment period under the Kyoto Protocol during COP 17 [47], it acknowledges that harvested wood products enhance the mitigation potential by carbon storage and substitution of fossil fuels by energetic and material use. This offers a substantial reduction of CO_2_ emissions [48-52]. In this comprising view the mitigation potential of forest management and timber utilisation offers far higher rates than the mere conservation of carbon stocks in forests [49,53-56].

## Conclusion

The use of CO_2_ flux measurements for determining sequestration rates and relating those to biological processes is a valid and important method, e.g. understanding the global carbon cycle and the determination of NPP. However, utilising of those results to quantify the amount of carbon stored in different forest carbon pools is subject to major uncertainties. Advice for good forest practice or political decision making becomes critical when these underlying uncertainties are ignored. Statements such as the “exponential growth of unmanaged forest in high ages” go without biological evidence and result in misleading advice for good practice and the efficient utilisation of the mitigation potential of forests.

However, an “unexpected” growth, compared to common yield tables, is recorded (e.g. [39]). Additional CO_2_ sequestration by increasing growth rates has been observed for managed as well as unmanaged forests in central Europe [33,35,40] and bears favourable potentials for the mitigation of climate change. Significant evidence exists that such additional growth is related to changed environmental conditions, i.e. increased nitrogen depositions, warmer temperatures and potentially CO_2_ fertilising effects [32,37,38]. The magnitude of its impact on growth, respectively biomass accumulation or carbon sequestration, depends on complementing conditions like former nutrient deficiencies, moisture and stand age. Still, those impacts equally affect managed as well as unmanaged forests. By that, it does not allow unidirectional conclusions against or in favour of forest management, e.g. for climate change mitigation. Moreover, the experience that older forests had the strongest relative increase in carbon sequestration should lead to the reconsideration of common management practises.

A more accurate assessment of the impact of management decisions on biomass growth and carbon sequestration could support a more suitable accounting of LULUCF, e.g. under the Kyoto Protocol. It could as well lead to better understanding about the importance of unmanaged and managed forest stands and acknowledge further ambitions for mitigation of future climate change, and justify respective incentives.

It is widely accepted that unmanaged stands or old-growth forests are of paramount importance for carbon storage and manifold further environmental services. Maintaining and enhancing the role of forests in the global carbon cycle needs to carefully compare the potential of different forest management strategies to mitigate climate change and to extend beyond a mere consideration of unmanaged, old-growth forests.

However, various authors underline the importance and existing advantages of forest management compared to unmanaged stands for the additional uptake of atmospheric CO_2_ and for the mitigation of climate change [10-12,22,57,58]. For a holistic view it is essential to widen the discussion and consider the dynamics and potential of carbon sequestration in forests beyond forest ecosystems and include the timber sector.

To summarize, those results support a more holistic view on forest management. While recognizing the multiple benefits of unmanaged, old-growth forests, sustainable forest management expands the mitigation potential beyond the mere sequestration of CO_2_ by photosynthesis resulting in forest growth. Given the larger growth and sequestration rates of managed forests and the sequestration and substitution effects of harvested wood products, it is obvious that future discussions should not focus on the abandonment of any forest management but on the selection of carbon-optimal forest management strategies. It must also be considered that abandonment of wood utilisation in some areas would inevitably lead to intensification in other areas – or increased wood imports from elsewhere – considering the demand for wood in central Europe for material or energetic utilisation.

Current discussions on optimal management strategies (rotation length, thinning interval etc.), are comprehensive – and potentially arguable under an inclusion of further goals than carbon sequestration compare [13,59-61]. While the observed relatively pronounced increase in carbon sequestration of old-growth forests could foster the extension of rotation periods, it must be considered that growth rates of these old and mature forests are rather low and their share in the forest area relatively small. Smaller relative changes in younger stands are most likely going to have a bigger net effect for the European forest carbon balance. In addition, traditional forest management concepts of increment-optimised maintenance of relatively high biomass stocks (“zuwachsoptimierte Vorratshaltung”) could be revitalized. By this the contribution of temperate forests to the mitigation of climate change could be improved by focusing forest management at mature (but not over-aging) stands with high growing stock volume. In this way, managed forest stands contribute to climate change mitigation at far higher rates than non-management of forests [62].

## Competing interest

The authors declare that they have no competing interest.

## Authors’ contributions

JK assembled the contribution, MK and DK contributed substantially. All authors have read and approved the final version of the manuscript.
